# MicroRNA-375-mediated regulation of ILC2 cells through TSLP in allergic rhinitis

**DOI:** 10.1016/j.waojou.2020.100451

**Published:** 2020-08-09

**Authors:** Xi Luo, Qingxiang Zeng, Shengbao Yan, Wenlong Liu, Renzhong Luo

**Affiliations:** Department of Otolaryngology, Guangzhou Women and Children's Medical Center, Guangzhou Medical University, Guangzhou, China

**Keywords:** Allergic rhinitis, HNECs, ILC2s, miR-375, AR, Allergic rhinitis, ILC2, Type II innate lymphoid cells, TSLP, Thymic stromal lymphopoietin, PCR, Real-time polymerase chain reaction, ELISA, Enzyme-linked immunosorbnent assay, HNECs, Human nasal epithelial cells

## Abstract

**Background:**

Studies have shown that the number and function of type II innate lymphoid cells (ILC2) in peripheral blood of allergic rhinitis (AR) children increased significantly. This study aims to evaluate the role of miR-375 in the regulation of the differentiation and function of ILC2 through both *in vivo* and *in vitro* studies.

**Methods:**

The expression of miR-375, thymic stromal lymphopoietin (TSLP) and the frequency of ILC2 were detected and compared between AR children and controls by real-time polymerase chain reaction (PCR), enzyme-linked immunosorbnent assay (ELISA) and flow cytometry, respectively. The miR-375 mimics or inhibitors were transfected into human nasal epithelial cells (HNECs), and the production of TSLP was detected by ELISA. HNECs and ILC2s were co-cultured to explore the role of miR-375 on ILC2s. AR mice models were established to prove the effect of miR-375 on ILC2s *in vivo*.

**Results:**

The expression of TSLP, miR-375, and the frequency of ILC2 were significantly higher in AR compared with controls. We found that the TSLP expression by HNECs were significantly higher when transfected with miR-375 mimics than in those transfected with miR-control and miR-375 inhibitor. In the coculture system, HNECs transfected with miR-375 mimics promote the type II cytokines production by ILC2, and this effect was blocked by anti-TSLP. Our results also showed that the miR-375 inhibitors attenuate allergic symptoms and production of type II cytokines in AR mice.

**Conclusions:**

Our findings suggest that miR-375-mediated regulation of ILC2 cells through TSLP, providing new potential treatment target for AR.

## Introduction

Allergic rhinitis (AR) is one of the most common chronic diseases in otorhinolaryngology. Clinical symptoms such as nasal obstruction, itching, sneezing, and runny nose caused by AR have a serious impact on the quality of life of children and consume huge social and medical resources.^1^

Type II innate lymphoid cell (ILC2) is a kind of innate lymphocyte with unique phenotype (Lineage^−^CD127^+^CRTH2^+^), which mainly distributes in adipose-related lymphoid tissue, intestine, lung, and skin. Under the stimulation of IL-25, IL-33, and TSLP from epithelial cells, ILC2 can secrete a large number of type II cytokines, such as IL-5, IL-13, and a small amount of IL-4.^2^ TSLP, mainly expressed by epithelial cells, is type 1 cytokine which is similar to interleukin 7 (IL-7).^3^ A recent study has confirmed that TSLP can regulate the function of ILC2 cells, promote the activation of ILC2 cells, and resist hormone therapy.^4^

MiRNA is an evolutionarily conserved small non-coding RNA, consisting of about 22 nucleotides.^5^ MiR-375 belongs to the highly conservative miRNA family and was first found to have the function of regulating insulin secretion in pancreatic tissue.^6^ In recent years, studies showed that miR-375 also plays important roles in Th2-related diseases and TSLP production.^7^, ^8^, ^9^ However, its role in ILC2 cells was not clear.

In this study, we first detected the expression of miR-375, TSLP, and ILC2 in AR children to confirm their correlation. Secondly, the expression of TSLP by allergen-stimulated airway epithelial cells was detected by transfecting mimic and inhibitor of miR-375. Finally, the activation of ILC2 and the pathological changes in allergic mice was detected.

## Methods

### Patients

Twenty-four AR children were enrolled from our medical center from July, 2018 to December, 2018. The inclusion criteria included: medical history, nasal symptoms, sensitization to allergens confirmed by skin prick test or specific IgE measurement. The atopic status was evaluated by a skin prick test (wheal diameter of at least 3 mm) and the detection of IgE (>0.35 kIU/L, Phadia, Uppsala, Sweden) specific to common inhalant allergens (dust mites, pollens, pets, molds, and cockroach, etc). All the AR children in this study were sensitive to *Dermatophagoïdes pteronyssinus* and/or *Dermatophagoïdes farinae*.

Children with positive test to allergens except for dust mites, asthma, anatomic abnormalities, or undergoing systemic steroids or having systemic immunologic disorders, and intercurrent treatment with β-blockers or oral corticosteroid treatment in the previous 2 months, were excluded. Twenty healthy controls were also enrolled. Our research was approved by the local committee (No. 20180104), and informed consent was obtained from the children's parents.

### Clinical evaluation

The symptoms and medications usage were recorded in patient diary and averaged in an 8-week observation period as described previously.^10^^,^^11^ The nasal symptoms, such as runny nose, sneezing, blocked nose, itchy nose, were scored as follows: 0 = no symptoms, 1 = slight symptoms, 2 = moderate symptoms, and 3 = severe symptoms. The daily medication score was calculated as the sum of medication administered at a particular day: cetirizine tablets (10 mg), 2 points/tablet; intranasal corticosteroid, 1 point/puff; leukotriene receptor antagonist, 1 points/tablet.

### Blood collection

Venous blood samples were obtained into Vacuette tubes from study subjects between 6:00 am and 8:00 am after an overnight fast. After centrifuging at 1000 g for 15 min at 4 °C, serum samples were separated and stored at −80 °C until assay. For ILC2 detection, peripheral blood mononuclear cells (PBMCs) were purified from fresh blood cells, as described earlier.^12^ Briefly, the blood was diluted using Dulbecco's Phosphate Buffered Saline with 2% Fetal Bovine Serum and layered on top of Lymphoprep carefully to reduce mixture of blood with Lymphoprep. After centrifugation at 800*g* for 20 min at room temperature, the upper plasma layer was discarded without disturbing the interface of plasma and Lymphoprep.

### Flow cytometry for ILC2

Lineage negative cells were enriched from PBMCs using FITC lineage cocktail (eBioscience, San Diego, CA) and FceRI (eBioscience). Cells were further stained with PE-conjugated CRTH2 (BD Bioscience, NJ) and PE-Cy7 conjugated CD127 (BD Bioscience, NJ). Human and mice ILC2s were identified as Lin^−^CRTH2^+^CD127^+^ and Lin^−^ST2^+^CD45^+^CD90.2^+^CD25^+^Sca1^+^ lymphocytes, respectively. Flow cytometry was performed by the Beckman flow cytometer machine (Beckman Coulter, Hercules, CA, USA).

### ILC2 sorting

As described above, the Lin^−^CRTH2^+^CD127^+^ lymphocytes were obtained using a MoFlo XDP cell sorter (Beckman Coulter, CA, USA). ILC2 was cultured in 96-well plate at a concentration of 2.5 × 10^5^ cells/ml in RPMI-1640, containing 10% FBS and 1% penicillin/streptomycin. The stimulating conditions were IL-25 (10 ng/ml), IL-33 (10 ng/ml), TSLP (10 ng/ml) and IL-2 (50 ng/ml). All cytokines and inhibitors were purchased from R&D system.

### Human nasal epithelial cell culture and coculture with ILC2

Human nasal epithelial cell line (HNECs) was obtained (ATCC, Rockville, MD, USA) and cultured in bronchial epithelial cell basal medium (BEBM, Lonza, Walkersville, MD, USA) at 37 °C in a 5% CO_2_-humidified chamber. After the cells were differentiated, they were incubated with 25 μg/mL *Dermatophagoides farinae* (Greer Laboratories, Lenoir, NC, USA) under different transfection status as described below. After 24 h of treatment, culture supernatants were collected and centrifuged at 500 rpm for 5 min to remove cell debris for subsequent analysis.

HNECs and ILC2s were co-cultured in Transwell chamber. The stimulating cytokines from R&D system company included IL-25 (10 ng/ml), IL-33 (10 ng/ml), TSLP (10 ng/ml), IL-2 (50 ng/ml), anti-TSLP (10 ng/ml), 100 ng/ml AG490 (JAK inhibitor). The treatment lasted 24 h at the presence of *Dermatophagoides farinae*.

### MiRNA mimic or inhibitor transfection

In different groups, 1 μM miR-375 mimics, miR-375 inhibitor, miR-375 control (Qiagen, Valencia, USA) were transfected in serum-free ACCELL medium (Thermo Science, Colorado, USA) for 1 h.

### Quantitative real-time PCR (qRT-PCR)

For miRNA, total RNA from whole blood was transcribed into cDNA reversely using Taqman microRNA reverse transcription kit. The Taqman microRNA assays (Invitrogen) was performed according to the manufacturer's instructions. The data were calculated using the 2^−ΔΔCt^ method and normalized to RNA U6 controls.

### Enzyme-linked immunosorbent assay (ELISA)

The levels of type II cytokines and TSLP were measured by ELISA kits (R&D systems, USA) according to the protocol provided by the manufacturer. The detection limits of the assays were as follows: IL-4, 0.22 pg/mL, IL-5, 3.9 pg/mL, IL-13, 125 pg/mL, TSLP, 31.2 pg/mL.

### Animal model

The study was approved by the local Animal Ethic Committee. Eight week-old male BALB/c mice intrapenitoneally injected with 100 μl of PBS containing 100 μg of OVA and 1.6 mg Al(OH)_3_ on day 0 and day 7. The mice were challenged nasally with 100 μg of OVA in 40 μL of PBS (20 μL per nostril) on day 14–18. For intervention experiment, the 100 nmol miR-375 mimics or inhibitors (GeneChem Company, Shanghai, China), or 10 μg/mL anti-TSLP (100 μl, R&D system) were given to mice intranasally 2 h before each OVA challenge. The mice were sacrificed next day of the last nasal challenge. Samples of the blood and nasal mucosa were collected for further experiments.

### Statistical analysis

The statistical analysis was performed using SPSS 17.0 software (IBM Corp., New York, NY). Data were expressed as mean ± SD and analyzed by the Kruskal-Wallis H test or the nonparametric Mann-Whitney *U* test. One-way analysis of variance with Tukey’ s post hoc test was used to determine the significance between more than two groups. A *P* < 0.05 was considered as statistically significant.

## Results

### Increased expression of TSLP, miR-375 and ILC2 in AR children

**Fig. 1:**
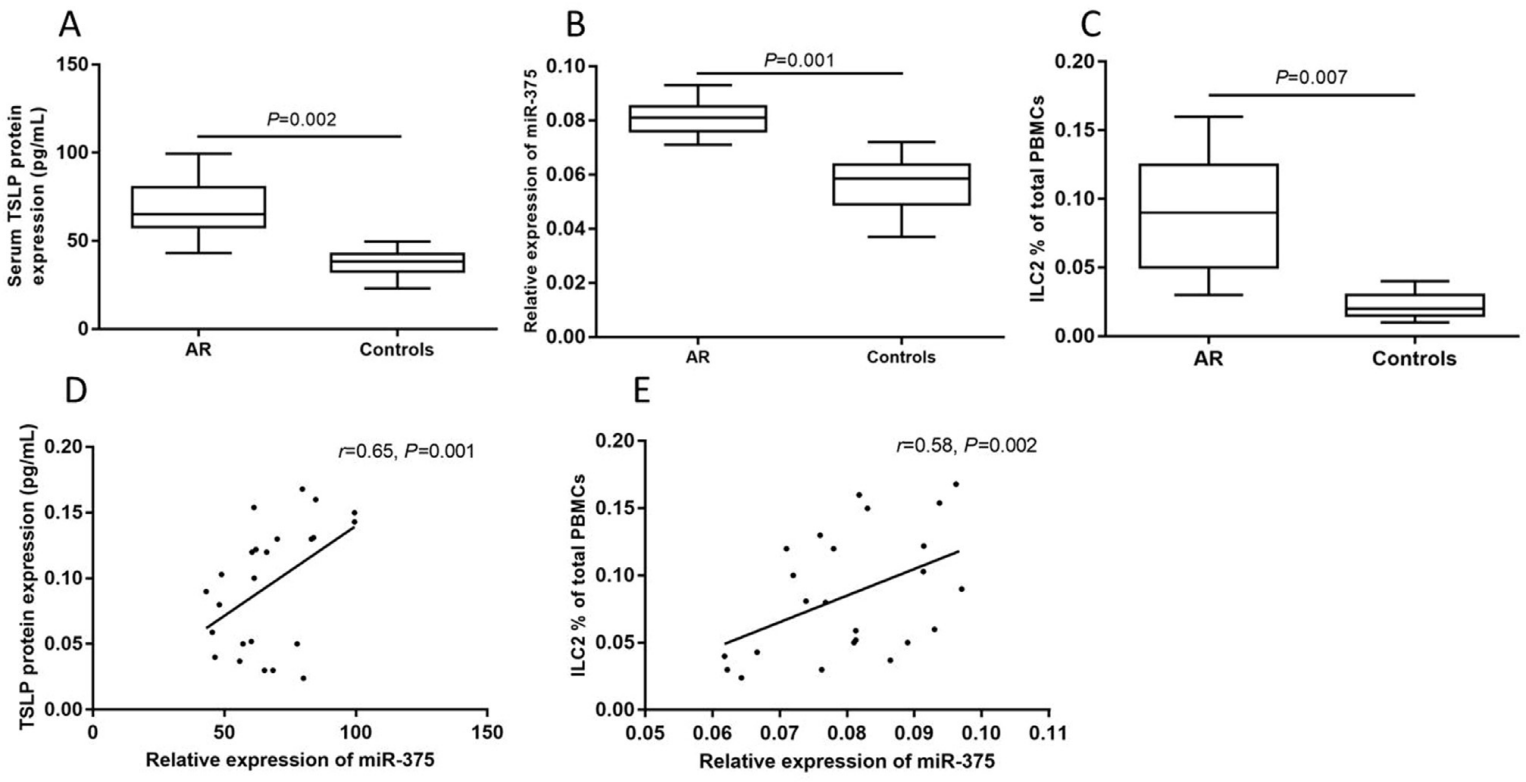
Elevated serum thymic stromal lymphopoietin (TSLP) (A), whole blood miR-375 (B), and type II innate lymphoid cells (ILC2) frequency (C) in peripheral blood mononuclear cells (PBMCs) in AR and the positive correlation between miR-375 and TSLP levels and ILC2 frequency (D,E) in children with AR

The demographic characteristics of subjects were summarized in Table 1. The serum TSLP levels were significantly higher in AR children compared with controls. The expression of miR-375 from whole blood and ILC2 frequency in blood were significantly elevated in AR children compared with controls. We also found that elevated miR-375 was positively correlated with TSLP levels and ILC2 frequency (Fig. 1).

**Table 1:** Demographic characteristic of AR and control children.

Groups	AR group	Control
Number	24	20
Sex (Male:Female)	11:13	11:9
Age (years)	12.3 (6–18)	11.8 (6–18)
Eosinophil cationic protein (ng/ml)	45.2 (31.0–137.0)^a^	10.9 (6.7–103.0)
Total IgE (IU/mL)	135.1 (41.2–601.1)^a^	20.9 (3.2–34.7)
Skin prick test-positive	24	0
Specific IgE to D1 (kIU/L)	9.8 (2.6–44.5)	<0.35
Specific IgE to D2 (kIU/L)	12.3 (1.8–36.7)	<0.35
TNSS score	8.2 (6–12)	–
Medication score	3.6 (2–5)	–

AR, allergic rhinitis; D1, *Dermatophagoïdes pteronyssinus*; D2, *Dermatophagoïdes farina*; TNSS, total nasal symptom score.

a Compared with control group, *P* < 0.05

### MiR-375 promotes the expression of TSLP by human nasal epithelial cell through JAK2-STAT3 pathway

**Fig. 2:**
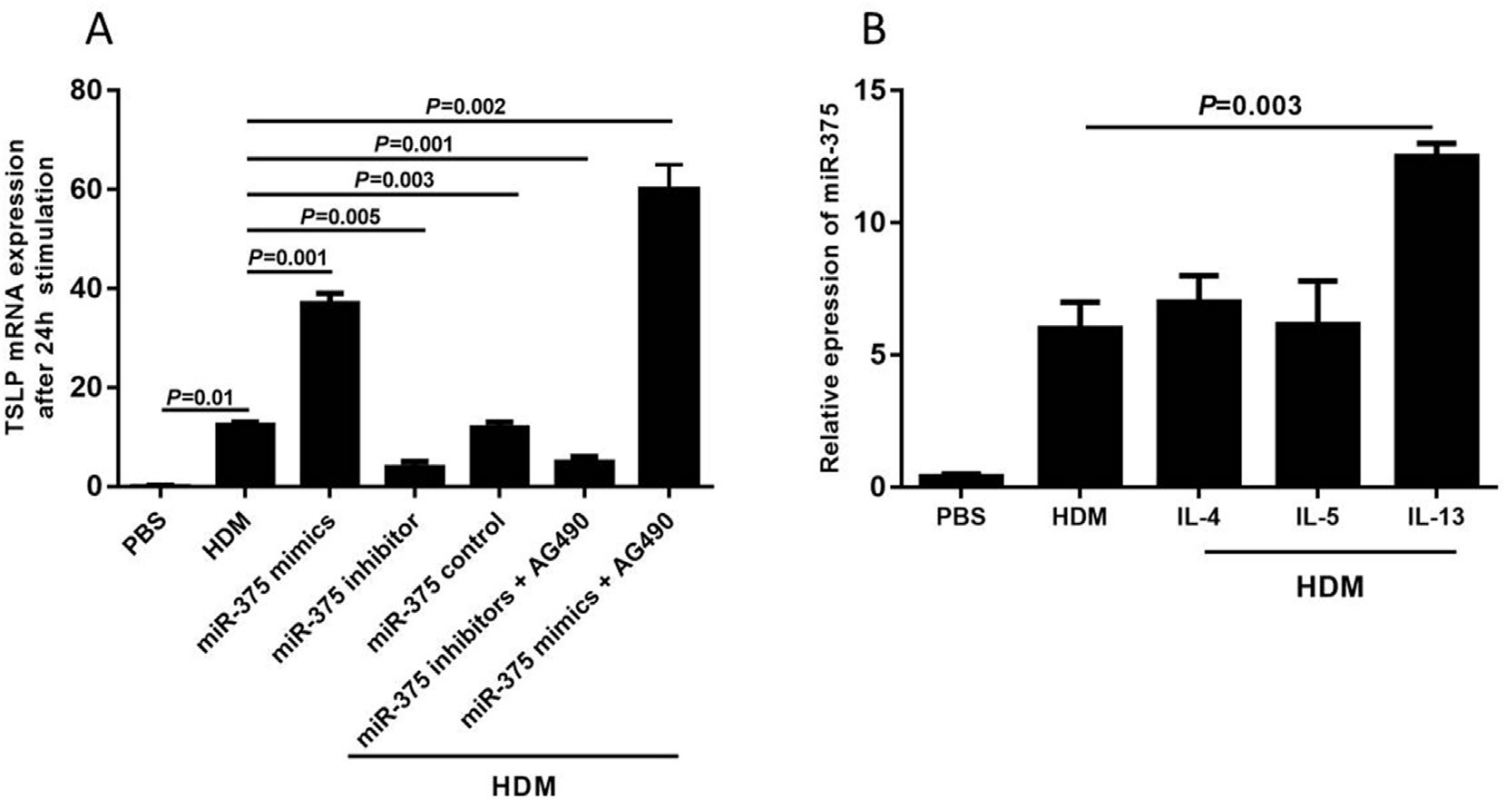
The expression of thymic stromal lymphopoietin (TSLP) by human nasal epithelial cell (HNECs) after transfected with miR-375 mimics, miR-control, miR-375 inhibitor or AG490 (JAK2-STAT3 inhibitor) in Figure A. The effect of Th2 cytokines on the expression of TSLP by HNECs was shown in Figure B. HDM, Dermatophagoides farinae

After HNECs were transfected with miR-375 mimics, the TSLP expression by HNECs were significantly higher than in those transfected with miR-control and miR-375 inhibitor. Moreover, this effect was significantly enhanced when JAK2-STAT3 inhibitor (AG490) was added, suggesting that the effect of miR-375 on TSLP was through inhibition of JAK2-STAT3 pathway. We also found that AG490 only restored the TSLP production partially without significance after transfected with miR-375 inhibitor. We also found that IL-13 and HDM could promote miR-375 expression (Fig. 2).

### MiR-375 promotes the expression of type II cytokines mediated by TSLP in coculture system of ILC2 and human nasal epithelial cell

**Fig. 3:**
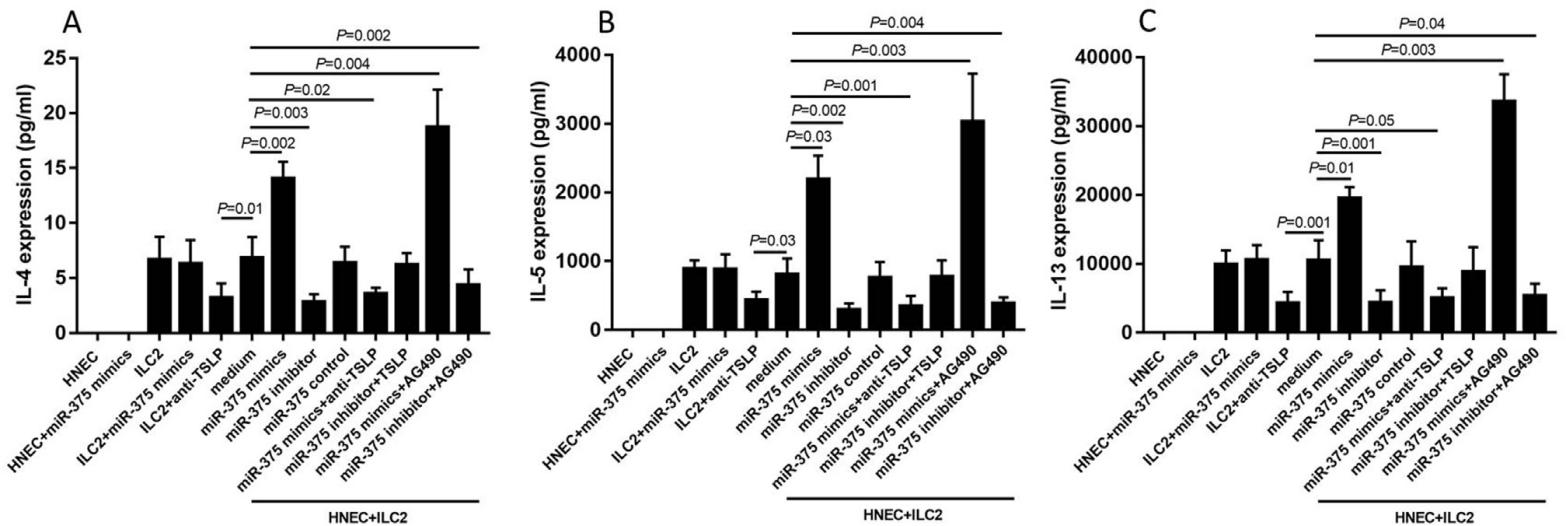
The expression of type II cytokines by type II innate lymphoid cells (ILC2) in the coculture system with human nasal epithelial cell (HNECs) in different combination of stimulators. AG490, JAK inhibitor. TSLP, thymic stromal lymphopoietin

In the coculture system, HNECs transfected with miR-375 mimics promoted the type II cytokines production by ILC2, which was blocked when anti-TSLP was added. Moreover, type II cytokines production was significantly enhanced when AG490 was added, suggesting that the effect of miR-375 on TSLP was through inhibition of JAK2-STAT3 pathway. We also found that HNECs transfected with miR-375 inhibitor decreased the type II cytokines production and the production of type II cytokines was recovered when TSLP was added. Moreover, AG490 only restored the type II cytokines production partially without significance after transfected with miR-375 inhibitor (Fig. 3).

### The miR-375 augments ILC2 responses in mice

**Fig. 4:**
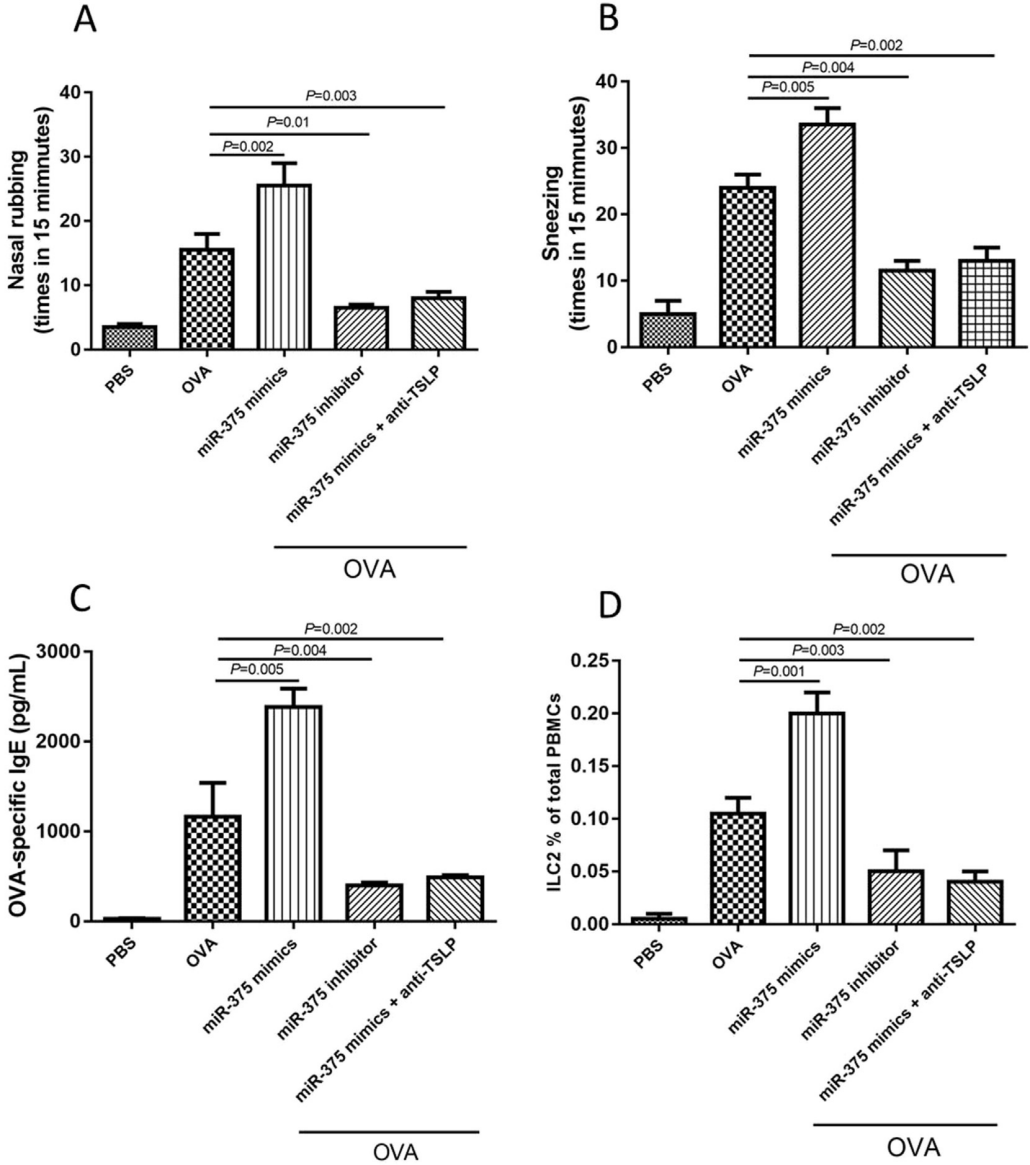
The ovalbumin (OVA)-specific IgE level, frequency of nasal rubbing and sneezing, and the number of type II innate lymphoid cells (ILC2) cells in peripheral blood mononuclear cell (PBMCs) in allergic mice treated with miR-375 mimics, miR-375 inhibitors or anti-TSLP. TSLP, thymic stromal lymphopoietin

We found that AR mice challenged with miR-375 presented with higher OVA specific IgE, higher frequency of nasal rubbing and sneezing, more ILC2 cells in PBMCs compared with control mice. Our results also showed that the miR-375 inhibitors and anti-TSLP attenuate production of type II cytokines and the number of ILC2 cells of PBMCs in AR mice (Fig. 4).

## Discussion

In this study, the regulatory effects of miR-375 on TSLP/ILC2 axis were explored. Our results confirmed that miR-375 can regulate the function of ILC2 by targeting TSLP.

The miR-375 was reported to be down-regulated in patients with Th2-associated diseases, such as atopic dermatitis and ulcerative colitis.^13^^,^^14^ Besides, up-regulated miR-375 can regulate IL-13–induced genes.^15^ However, our data showed that peripheral blood miR-375 was up-regulated significantly and correlated with frequency of ILC2, the expression of type II cytokines and TSLP in peripheral blood in AR, providing evidence that miR-375 may be involved in the regulation of ILC2 through TSLP in AR. These data suggest that the mechanisms of different miR-375 activity in different types of Th2-associated diseases need further exploration.

To confirm the direct effect of miR-375 on TSLP expression, we transfected miR-375 mimics and inhibitors into HNECs. We found that the TSLP expression were significantly up-regulated when transfected with miR-375 mimics. This effect was significantly enhanced when AG490 was added, suggesting that the effect of miR-375 on TSLP was through inhibition of JAK2-STAT3 pathway. Consistently, miR-375 has been found to inhibit Helicobacter pylori-induced gastric carcinogenesis by blocking JAK2-STAT3 signaling.^16^ JAK2 has been also proved to be a potential target of miR-375 in various studies.^17^^,^^18^ However, whether other pathways were involved in the miRNA-375's effect on TSLP needed further exploration.

To mimic the real state, we cultured HNECs and ILC2 together. We found that HNECs transfected with miR-375 mimics promote the type II cytokines production by ILC2, especially IL-13. However, this effect was significantly blocked by anti-TSLP, suggesting that miR-375 mediated type II cytokines production by ILC2 through regulation of TSLP. Consistently, miR-375 was also found to regulate production of thymic stromal lymphopoietin (TSLP) in HT-29 cells.^19^^,^^20^

We also found that HDM and IL-13 could promote miR-375 expression, which forming a positive loop. Consistently, Biton's study found that IL-13 stimulation induces miR-375 expression in HT-29 human colon adenocarcinoma cells.

In the mice model, miR-375 challenge contributed to more severe symptoms and ILC2 inflammation compared with control mice. Our results also showed that the miR-375 inhibitors and anti-TSLP inhibited the production of type II cytokines and the number of ILC2 cells in PBMCs, which confirmed the *in vitro* results.

In summary, our findings showed that microRNA-375-mediated regulation of ILC2 cells through TSLP and JAK2-STAT3 signaling pathway, providing new potential treatment target for AR.

## Support statement

This study was supported by grants from the National Natural Science Grant of China (No.81600785, No. 81700892, No.81970861), the 10.13039/100015351Pearl River S&T Nova Program of Guangzhou (No.201710010085), Key Clinical Speciality of Guangzhou Women and Children's Medical Center, Grant of Institute of Pediatrics of Guangzhou Women and Children's Medical Center (YIP-2016-022, Pre–NSFC–2018-005).

## Ethics approval and consent to participate

The study protocols were approved by local ethics committee boards and written informed consent was obtained.

## Availability of data and materials

The datasets used and/or analyzed during the current study are available from the corresponding author on reasonable request.

## Author contributions

Dr Wenlong Liu and Renzhong Luo conceptualized and designed the study, drafted the initial manuscript, and approved the final manuscript as submitted. Dr Xi Luo, Qingxiang Zeng, Shengbao Yan collected the sample, performed the experiment, data collection and statistics, reviewed and revised the manuscript, and approved the final manuscript as submitted.

## Consent for publication

All authors agreed to publication of the work.

## Disclosure of potential conflict of interest

The authors declare that they have no relevant conflicts of interest.
